# Anti-Quorum Sensing Activity of 12 Essential Oils on *chromobacterium violaceum* and Specific Action of *cis-cis-p*-Menthenolide from Corsican *Mentha suaveolens* ssp. *Insularis*

**DOI:** 10.3390/molecules23092125

**Published:** 2018-08-23

**Authors:** Jean-Pierre Poli, Elodie Guinoiseau, Dominique de Rocca Serra, Sylvain Sutour, Mathieu Paoli, Félix Tomi, Yann Quilichini, Liliane Berti, Vannina Lorenzi

**Affiliations:** CNRS UMR 6134 SPE, Université de Corse, BP 52, 20250 Corte, France; poli_jp@univ-corse.fr (J.-P.P.); deroccaserra_d@univ-corse.fr (D.d.R.S.); sylvain.sutour@unine.ch (S.S.); paoli_m@univ-corse.fr (M.P.); tomi_f@univ-corse.fr (F.T.); quilichini_y@univ-corse.fr (Y.Q.); berti_l@univ-corse.fr (L.B.); lorenzi_v@univ-corse.fr (V.L.)

**Keywords:** *Chromobacterium violaceum*, quorum sensing, essential oils, *cis-cis-p*-menthenolide, biofilms

## Abstract

Quorum sensing (QS) is a bacterial communication mechanism used to express various survival or virulence traits leading to enhanced resistance. *Chromobacterium violaceum* is a commonly used strain that highlights anti-QS action of bioactive substances. Here, we wanted to see if 12 selected essential oils (EO) could exert anti-QS activity. We measured the sublethal minimal QS inhibitory concentration (MQSIC) by assessing violacein production of *C. violaceum* along with bacterial growth. To confirm the QS disruption, we also proceed to surface bacterial observations using scanning electron microscopy (SEM). We showed that *cis-cis-p-*menthenolide extracted and isolated from a plant endemic to occidental Mediterranean Sea islands, *Mentha suaveolens* ssp. *insularis*, acts as an inhibitor of violacein production and biofilm formation. Measured MQSIC was much lower than the minimal inhibitory concentration (MIC): 0.10 mg·mL^−1^ vs. 3.00 mg·mL^−1^. Moreover, disturbance of QS-related traits was confirmed by the degradation of *C. violaceum* biofilm matrix. There is a clear structure–activity relationship between *cis-cis-p-*menthenolide and anti-QS activity. Indeed, its isomer molecule (mintlactone) exerts a poor anti-QS action. These results indicate that inhibition of violacein production and biofilm formation by *cis-cis-p-*menthenolide might be related to a disruption in the QS mechanism.

## 1. Introduction

Nowadays, bacterial resistance to antibiotics is a major public health issue. Development of new antibiotics with new modes of action is very limited. One potential strategy to override this problem is to restore antibiotic activity. Inhibition of quorum sensing (QS) may be a suitable solution.

QS is a bacterial cell-to-cell communication pathway. This specific signal-response system relies on the synthesis, release, and uptake of specific molecules known as autoinducers [1]. When a specific concentration is reached in the outer medium, which is correlated to population concentration, autoinducers are the starting point to trigger and synchronize QS-related behaviors or traits expression. QS regulation is mainly based on the production of three types of molecules [2]:

- *N-acyl* homoserine lactones, produced by Gram-negative bacteria, used to monitor the population density and thus trigger the process of QS-mediated gene expression;
- Oligopeptides or autoinducing peptides, which are the main autoinducers in Gram-positive bacteria. They are exported in the outer medium, modified post-translationally and retaken by other cells by a two-component regulatory system;
- Autoinducer 2 (AI-2), used for interspecies communication between Gram-negative and Gram-positive bacteria.

*Chromobacterium violaceum* is a well-known bioindicator used to find substances that can block the QS mechanism [3]. It is a gram-negative β-proteobacterium commonly found in soil and water of tropical and subtropical regions [4,5]. QS influences various traits of *C. violaceum* such as elastase production [6], biofilm formation [7], chitinolytic activity [8], cyanide [9], or violacein production [7]. Violacein is an antibiotic, purple pigment produced under control of the *LuxR*–*LuxI* homolog system, *CviR*–*CviI* and the cognate molecule *N*-Hexanoyl-l-homoserine lactone [10,11,12]. Because it is easily observable and quantifiable, it is used as a tool to measure the impact of various substances on QS.

The search for QS disruptor could lead to new opportunities in fighting bacterial colonization. An anti-pathogenic action could be useful knowing the biofilm formation problems retrieved on catheters during the hospital treatment-time that lead to complication or even nosocomial infections.

But an anti-biofilm molecule could also found its use in the industry. Indeed, it could be used as an antifouling product as biofilm formation is the first necessary step of the biofouling formation that corresponds to the colonization of surface by macroorganisms such as, marine invertebrate larvae or macroalgae spores. Although it is a natural process, its formation on underwater material such as pipelines, port facilities or ship hulls can lead to enormous economic and ecologic damage [13,14].

Essential oils (EOs) are complex natural mixtures of volatile secondary metabolites and fragrant substances biosynthesized by plants. EOs are isolated by steam/hydro-distillation or by cold expression [15]. They are known to have multiple biological activities, such as antibacterial, antiviral, antifungal, antioxidant, wound healing, anti-inflammatory or immunomodulatory properties [16,17,18,19]. Given the wide spread of bacterial resistance, disrupting the QS mechanism seems to be an interesting alternative. For pathogenic organisms, virulence regulation is a crucial point in the infection process. Indeed, QS appears as a key factor, allowing, for example, bacteria population to growth substantially before expressing virulence factors, leading afterward to an overwhelming of the host defenses [20]. Unlike antibiotics, whose purpose is to destroy bacteria, which leads to the appearance of resistance, attenuation of the virulence via a QS disruptor might offer a new alternative for the development of new antimicrobial molecules; even if there are no actual proof that it’ll reduce the risk of resistance apparition [21]. Some EOs like Mandarin or Marjoram have already been tested in the food industry and are known to have an anti-QS effect by preventing biofilm formation [22,23]. 

The purpose of this study was to investigate the anti-QS properties of 12 EOs on *C. violaceum*, which are known to exert several antibacterial activities [24,25]. To achieve this goal, we investigated one of the major molecules putatively responsible for the anti-QS activity, finding *cis-cis-p-*menthenolide was the most effective. 

## 2. Results and Discussion

### 2.1. Inhibition of Violacein Production

Twelve EOs were tested for their anti-QS activity on *C. violaceum*. Their effects on the production of QS-related violacein pigment were evaluated along with bacterial density counts. We measured their minimal inhibitory concentration (MIC) and the minimal QS inhibiting concentration (MQSIC), which is the minimal EO concentration that inhibits the violacein production of *C. violaceum* by at least 50% (Table 1).

Tested EOs showed a range of antimicrobial activities. According to Djabou et al. [24], EOs can be classified in three categories according to their antimicrobial activity. *C. violaceum* is not sensitive to *Cedrus atlantica* EO with a MIC value of 50.00 mg·mL^−1^. It is dominated by α-pinene (55%), a hydrocarbon molecule known to be less active than the oxygenated molecules [15]. The strain is moderately sensitive to *Helichrysum italicum*, *Citrus limon* and *Mentha suaveolens* ssp. *insularis* with respective MIC values of 12.50 mg·mL^−1^, 6.00 mg·mL^−1^ and 3.00 mg·mL^−1^. Lastly, *C. violaceum* appears to be sensitive to *Citrus clementina*, *Lavandula stoechas*, *Myrtus communis* (MIC values 1.50 mg·mL^−1^); *Eucalyptus polybractea*, *Calamintha nepeta* ssp. *nepeta*, *Cymbopogon flexosus, Foeniculum vulgare* (MIC values 0.80 mg·mL^−1^) and *Xanthoxylum armatum* (MIC value 0.40 mg·mL^−1^). For this last group, we retrieved EOs from which some major compounds are already known to exert antimicrobial activity, such as 1,8-cineole [26], fenchone [27], citral (geranial and neral) [28], cryptone [29], camphor and linalool [30].

*C. violaceum* QS was disturbed differently depending on the used EO. The MQSIC for *Cedrus atlantica* is high (6.00 mg·mL^−1^), thus it is not a relevant anti-QS EO. The EO of *Helichrysum italicum*, with MQSIC value of 0.80 mg·mL^−1^, followed by both *Citrus* EOs (0.40 mg·mL^−1^) are significantly lower but still a little high in comparison to the upcoming group. Next, there is a larger group of five EOs with MQSIC values of 0.20 mg·mL^−1^. Even if EOs action on QS is not widely studied, some essential oils exerted similar effect on *C. violaceum* QS. For example, the MQSIC values of Rosemary and Tea Tree EOs can be compared to this last group (0.21 mg·mL^−1^) [31]. At these concentrations, they are considered active against QS. Next are *Mentha suaveolens* ssp. *insularis* and *Myrtus communis* with MQSIC values of 0.10 mg·mL^−1^. Finally, the lowest MQSIC value was found for *Eucalyptus polybractea* (0.05 mg·mL^−1^). Colombian *Lippia alba* EOs, extracted from the whole plants, also had anti-QS activity. MQSIC of the EO from the Santander region is 0.09 mg·mL^−1^, which is similar to *Mentha suaveolens* ssp. *insularis* and *Myrtus communis* [32]. However, the isolation methods were different, which may have affected the concentration of the distillated molecules. The most active ones were extracted by microwave-assisted distillation. This method is known to produce a higher ratio of oxygenated compounds [27], but is different from the usual steam distillation method, which was used to produce EOs tested in our work.

We choose to evaluate the MQSIC/MIC ratio using the dilution values (Table 1). The higher the ratio, the better the anti-QS activity will be. *Xanthoxylum armatum* appears to be the least efficient EO. The observed reduction of violacein production is more likely to be due to an antimicrobial action than an anti-QS effect. *Calamintha nepeta* ssp. *nepeta* and *Citrus clementina* EOs only have a ratio value of 4, followed by both *Cedrus atlantica* and *Lavandula stoechas* with a value of 8. Such low ratio values show, again, that the MQSIC and MIC dilutions are very close. With a MQSIC/MIC value of 16, *Citrus limon*, *Eucalyptus polybractea*, *Helichrysum italicum* and *Myrtus communis* have an effective action on *C. violaceum* QS. Major compounds of those EOs have not, to our knowledge, been tested in anti-QS assays. But, as previously seen, Rosemary and Tea Tree EOs seem to exert anti-QS activity. Their major compounds are camphor (≈30%), 1,8-cineole (≈14%) [33] and terpinen-4-ol (≈29%), 1,8-cineole (≈17%) [34]. While 1,8-cineole is one of the major compounds of *Eucalyptus polybractea* and *Myrtus communis*, it was not found to be clearly responsible for anti-QS activity.

Only *M. suaveolens* ssp. *insularis* EO has an even higher MQSIC/MIC ratio of 32, making this EO the most attractive in terms of anti-QS activity. Since the sublethal anti-QS dilution is very different from the MIC one, this EO has the ability to inhibit preferentially the QS mechanism instead of inhibiting cell growth.

To find molecule(s) that could be responsible for the anti-QS activity of *M. suaveolens* ssp. *insularis* EO (Table 2), we tested both its major compounds: pulegone and *cis-cis-p-*menthenolide.

Pulegone is the most abundant compound of this EO, which is already know to exert an antimicrobial activity [35]. Experimentation on the commercial molecule showed that its MQSIC/MIC ratio value is 4, which is lower than the EO value by itself (32) and thus supporting its antibacterial action.

We then evaluated the anti-QS activity of *cis-cis-p-*menthenolide that we compared to the activity of mintlactone, its commercial isomer. This comparison was done to evaluate the structure-activity relationship of both molecules.

*cis-cis-p-*menthenolide((3aS,6S,7aS)-6-methyl-3-methylidenehexahydro-1-benzofuran-2(3H)-one)), a methylidene lactone bearing the menthane skeleton, is the second major component of this essential oil (27.3%). Nonetheless, this molecule is found in higher concentrations in the hydrolate extract (67.3%) [36]. As a water-soluble molecule, it is more likely to be found in this fraction of the plant hydrolate. For this reason, the hydrolate extract was used to isolate the molecule. *cis-cis-p-*menthenolide has a MQSIC/MIC ratio of 64, which is the highest found in our study, proving the anti-QS effect of that molecule. On the other hand, the mintlactone MQSIC/MIC ratio (8) is only slightly higher than pulegone (Table 2). Thus, its action can be considered more as antimicrobial than anti-QS. *cis-cis-p-*menthenolide and mintlactone exhibit the α,β unsaturated lactone substructure. However, they only differ by the double bond position (intracyclic or exocyclic), which is probably responsible for the difference in anti-QS activity between *cis-cis-p-*menthenolide and mintlactone. 

Since Gram-negative bacteria use acyl-homoserine lactone as diffusible signal molecules for QS, we can suppose that the lactone skeleton of those molecules could be responsible for interfering with and disrupting the QS mechanism in *C. violaceum*. These results highlight the disruptive role of *cis-cis-p-*menthenolide on violacein production and possibly on the QS system.

As this study was about the primary anti-QS mode of action of tested EOs, it’ll be interesting, in the future, to discover how *cis-cis-p-*menthenolide is interfering with QS. We could think about three main targets, acyl-homoserine-lactone synthase (*CviI*), the lactone itself (*N*-Hexanoyl-l-homoserine), or the transcriptional regulator (*CviR*). Knowing the homology found between lactone structure of *cis-cis-p-menthenolide* and *N*-Hexanoyl-l-homoserine, we might think to a competition between those two compounds. This could lead to a blocking in the process of gene expression normally induced by the *CviR*-*N*-Hexanoyl-l-homoserine binding process, but this will need to be tested through the use of fluorescent quenching.

### 2.2. Anti-Biofilm Action

**Figure 1:**
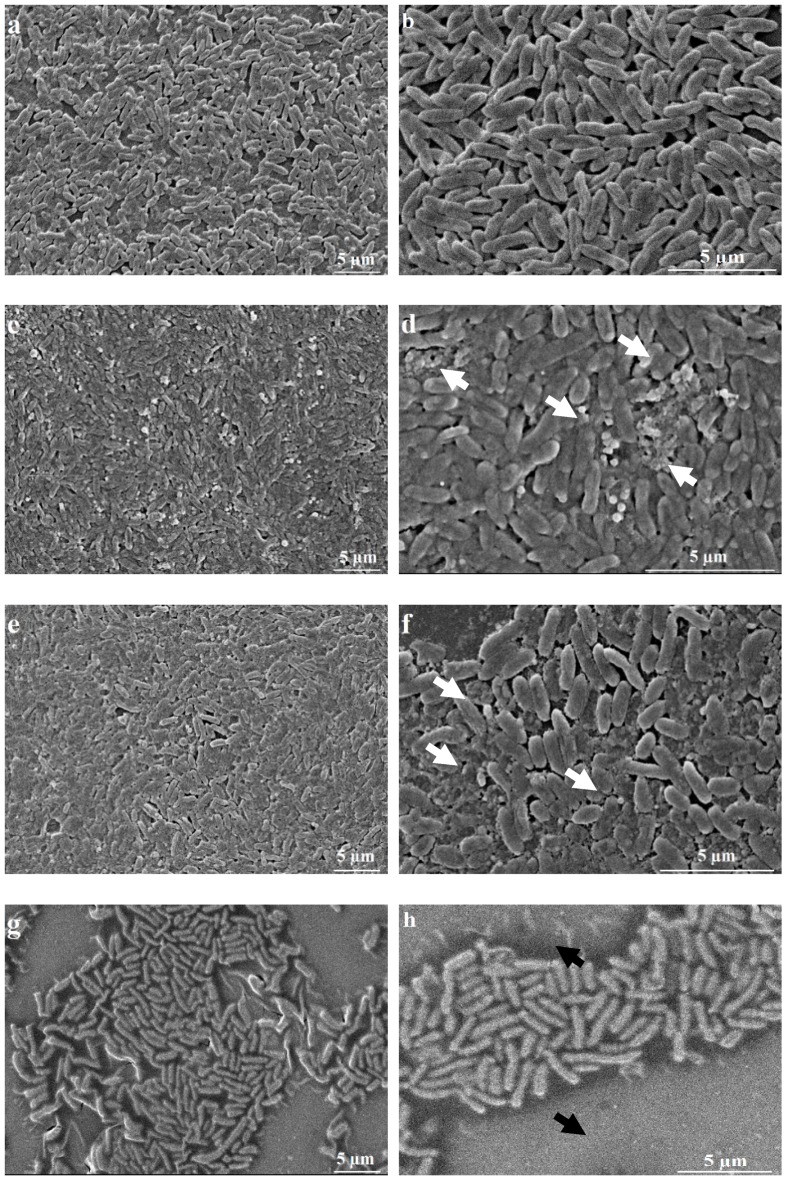
SEM images of *C. violaceum* growth in normal conditions (**a**,**b**), in the presence of *M. suaveolens* ssp. *insularis* at MIQSC (**c**,**d**), *cis-cis-p*-menthenolide at MIQSC (**e**,**f**) and mintlactone at MIQSC (**g**,**h**). White arrows in (**d**,**f**): biofilm matrix degradation; black arrows in (**h**): zones without bacterial development. Scale 5 µm.

To confirm the anti-QS activity of *cis-cis-p-*menthenolide, we choose to evaluate the inhibition of biofilm formation by scanning electron microscopy because it is mediated by QS in *C. violaceum*. Biofilm formation is a multistage process that starts with bacterial adhesion to a surface followed by the production and accumulation of an extracellular polymeric matrix [37]. The action of *M. suaveolens* ssp. *insularis* EO, *cis-cis-p-*menthenolide and mintlactone on biofilm formation are shown in Figure 1.

In standard conditions, *C. violaceum* grows in a very dense and homogenous biofilm (Figure 1a,b). When *M. suaveolens* ssp. *insularis* EO (Figure 1c,d) is used at its MQSIC, bacterial density is similar to the control condition, but the matrix seems to be degraded and fragmented as shown in the Figure 1d. The same can be said when assessing *cis-cis-p-*menthenolide (Figure 1e,f), with even more disaggregation of the matrix. On the contrary, when mintlactone is used at its MQSIC (Figure 1g,h), there is visible reduction of the population density. This observation seems to be in agreement with the MQSIC/MIC ratios in Table 2, where we show this molecule has no influence on the QS but rather exerts a direct action on the bacterial cell.

We clearly showed, in the first part of the study, that *cis-cis-p-*menthenolide action lead to a strong decrease in violacein production, thus without impacting bacterial density which is implying a disruption of the QS. So, visible results of its action on *C. violaceum* biofilm matrix appear as one other proof of the implied action against QS.

Further investigations could be undertaken in the future to confirm this hypothesis. For example, we could determine the chitinolytic activity of *C. violaceum*. Indeed, when in presence of chitin in a low nutrient environment, *C. violaceum* can, under QS control, induce chitinase production [8]. 

## 3. Materials and Methods

### 3.1. Bioactive Substances

Twelve EOs were tested. Hydro-distillations were made from various parts of the plants of interest: the leaves for *Mentha suaveolens* ssp. *insularis* and *Eucalyptus polybractea,* young leaves and green twigs for *Citrus clementina* and *Citrus limon*, aerial parts for *Myrtus communis, Cedrus atlantica*, *Calamintha nepeta* ssp. *nepeta* and *Cymbopogon citratus*, flowering tops for *Helichrysum italicum*, *Lavandula stoechas*, seeds for *Foeniculum vulgare* and dried fruits for *Xanthoxylum armatum*. They were mainly supplied by U Mandriolu Sarrola Carcopino (Corsica, France). Essential oil of *Mentha suaveolens* ssp. *insularis* was isolated from aerial part (leaves) of the plant by hydro-distillation in our laboratory.

Plant materials were dried one day at room temperature, away from direct sunlight, and submitted to water-distillation (3 h) using a Clevenger-type apparatus (yields: 0.14% *w/w*). The combined EO (10 mL) and the hydrolate (10 L) from the same distillation were stored in the dark at 4 °C. Three portions (3.3 L each) of the hydrolate were extracted with diethyl oxide (3 × 1 L) and the combined organic layer was dried over MgSO_4_. The solvent was removed under reduced pressure yielding the hydrolate extract (HE, 3.8 g).

Mintlactone and pulegone were purchased from Sigma-Adrich™ (St. Louis, MO, USA).

All products were solubilized in dimethyl sulfoxide (DMSO, Sigma-Aldrich™). Antimicrobial activity of the DMSO solvent was tested beforehand and showed no effect on bacterial growth at the tested concentrations.

### 3.2. Gas Chromatography (GC) Analyses

Analyses were performed on a Clarus 500 PerkinElmer (PerkinElmer, Courtaboeuf, France) system equipped with a FID and two fused-silica capillary columns (50 m × 0.22 mm, film thickness 0.25 µm), BP-1 (polydimethylsiloxane) and BP-20 (polyethylene glycol). The oven temperature was programmed from 60 °C to 220 °C at 2 °C/min and then held isothermal at 220 °C for 20 min; injector temperature: 250 °C; detector temperature: 250 °C; carrier gas: hydrogen (1 mL/min); split: 1/60; injected volume: 0.5 µL. The relative proportions of the oil constituents were expressed as percentages obtained by peak-area normalization, without using correction factors. Retention indices were determined relative to the retention times of a series of n-alkanes (C_7_–C_28_) with linear interpolation (Target Compounds software from PerkinElmer).

#### Gas Chromatography-Mass Spectroscopy (GC-MS) Analyses 

The EOs were analyzed with a PerkinElmer TurboMass detector (quadrupole), directly coupled to a PerkinElmer Autosystem equipped with a fused-silica capillary column (50 m × 0.22 mm i.d., film thickness 0.25 µm), BP-1 (dimethylpolysiloxane). Carrier gas, helium at 0.8·mL/min; split: 1/60; injection volume, 0.5 µL; injector temperature, 250 °C; oven temperature programmed from 60 °C to 220 °C at 2 °C/min and then held isothermal (20 min); ion source temperature, 250 °C; energy ionization, 70 eV; electron ionization mass spectra were acquired over the mass range 40–400 Da.

### 3.3. Chromatographic Fractionation of the Hydrolate Extract

Hydrolate extracts were chromatographed using a Grace Reveleris^®^ flash chromatography system using a linear gradient pentane/chloroform/methanol. The F19 fraction eluted with 20% chloroform in pentane contains 2.0813 g of *cis-cis-p*-menthenolide with a purity of 95% [36,38,39].

### 3.4. Bacterial Strains and Growth Conditions

*Chromobacterium violaceum* wild-type strain (CIP 103350T) was obtained from the Institut Pasteur’s Biological Resource Center (Paris, France). The strain was grown under aerobic condition in trypticasein soy (TS) broth and agar (Conda™, Torrejón de Ardoz, Spain), incubated at 30 °C for 24 h.

### 3.5. Anti-QS Assay: Violacein Quantification and Measurement of Cell Growth

The anti-QS activity of Corsican EOs was assessed on *C. violaceum* (10^6^ CFU·mL^−1^). Their concentrations ranged from 50.00 mg·mL^−1^ (dilution: 1) to 1.25 × 10^−2^ mg·mL^−1^ (dilution: 4096) and were obtained by a two-fold serial dilution method. *C. violaceum* was incubated in culture tubes containing TS broth at 30 °C on an orbital shaker (Stuart™, Stone, UK). Violacein production was quantified using the Choo, Rukayadi, & Hwang protocol [18]. Briefly, 1 mL of overnight culture of each culture tube was centrifuged at 13,000 rpm for 10 minutes to precipitate the pigment. The pellet was resuspended in 1 mL of DMSO and homogenized by pipetting. Violacein absorbance at 585 nm was measured by an UV-visible spectrophotometer (Jasco™, Oklahoma City, CA, USA). The control samples consisted of incubating *C. violaceum* in TS broth without the addition of bioactive substances. Each test was performed in triplicate. Percentages of violacein production were expressed using the following formula:

(absorbance of tested concentration at 585 nm/absorbance of the control at 585 nm) × 100(1)

**Figure 2:**
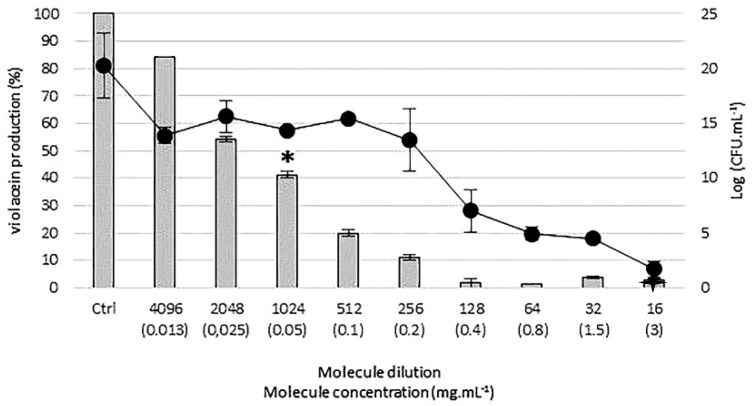
Effect of increasing molecule concentrations on *Chromobacterium violaceum* growth (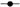) and violacein production (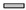). The example of *cis-cis-p-*menthenolide was used to show how MIC (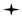) and MQSIC (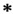) were determined.

Since a reduction of violacein production can be due to either blocking the QS mechanism or inhibiting bacterial growth [40], the bacterial concentration was evaluated in parallel: 10-fold serial dilutions were performed from each culture tube; 100 µL of each dilution was then plated on TS agar dishes and incubated at 30 °C for 24 h. Colonies were then counted and expressed as Log CFU·mL^−1^. This assay also allows us to determine the minimal inhibitory concentration (MIC), which is the minimal dilution where at least 90% of the bacterial population has not grown compared to the control (when growth is <2 Log CFU·mL^−1^ according to method sensitivity). According to Djabou et al. (2013) [24], a strain is considered as not sensitive for MIC value between 50.00 or above to 25.00 mg·mL^−1^, moderately sensitive for value between 12.50 and 3.00 mg·mL^−1^ and sensitive for value between 2.00 and 0.40 mg·mL^−1^. Finally, the minimum QS inhibitory concentration (MQSIC) was defined as the effective concentration of EOs or bioactive agents which for at least 50% of the violacein production was reduced [41] in absence of significant effect on cell growth (Figure 2). Each test was performed in triplicate. 

### 3.6. Anti-Biofilm Assay: Scanning Electron Microscopy

Scanning electron microscopy (SEM) was used to observe bacterial behavior in solution with EOs and molecules samples. For this purpose, 7.5 µL of each sample dilution were placed in a 96-well microplate containing 142.5 µL of a 0.5 McFarland standard of *C. violaceum* diluted to reach 10^6^ CFU·mL^−1^. A plastic lid with 96 identical pegs protruding downward (MBEC™ High Throughput (HTP) Assay Innovotech Inc., Edmonton, AB, Canada) was placed on top. These pegs allow bacterial development in a biofilm. The microplates were shaken at 30 °C for 36 hours. The pegs were immersed in a 2.5% glutaraldehyde (Electron Microscopy Science™, Hatfield, PA, USA) fixation solution in sodium cacodylate (Sigma-Aldrich™, St. Louis, MO, USA) buffer 0.1 M (pH 7.2) for 2 to 3 h at 4 °C. They were washed in two successive 10 min baths of sodium cacodylate buffer, dried for 120 h, and then coated with gold/palladium sputter coating (Quorum Technologies SC7640, Lewes, UK). The pegs were then examined under a scanning electron microscope (Hitachi™ S3400N, Tokyo, Japan) with an accelerating voltage of 5 or 10 kV. Each test was performed in triplicate and the images are representative of the bacterial distribution on the whole peg surface.

## 4. Conclusions

In summary, *cis-cis-p-*menthenolide, a *p*-menthane exhibiting a characteristic exocyclic α,β unsaturated lactone substructure is of specific interest because it is only found in the endemic to the occidental Mediterranean Sea islands *Mentha suaveolens* ssp. *insularis*. In this study, it appears that *cis-cis-p-*menthenolide, extracted from Corsican *Mentha suaveolens* ssp. *insularis*, seems to act as a quencher of the QS signaling pathway. Its sublethal MQSIC dilution of 1024 (0.05 mg·mL^−1^) had no bactericidal effect, but considerably reduced violacein production. Moreover, anti-QS action was enlightened by scanning electron micrographies where biofilm matrix appears to be altered without affecting bacterial density.

## Figures and Tables

**Table 1:** Determination of anti-QS activities of tested essential oils.

Essential Oils	MIC Value (mg·mL^−1^)/MIC Dilution	MQSIC Value (mg·mL^−1^)/MQSIC Dilution	MQSIC/MIC Ratio	Major Compounds
*Mentha suaveolens* ssp. *insularis*	3.00 (16)	0.10 (512)	32	pulegone (44.4%), *cis-cis-p-*menthenolide (27.3%)
*Citrus limon*	6.00 (8)	0.40 (128)	16	limonene (66.4%), γ-terpinene (10.1%)
*Eucalyptus polybractea*	0.80 (64)	0.05 (1024)	16	*p*-cymene (25.5%), cryptone (11.42%)
*Helichrysum italicum*	12.50 (4)	0.80 (64)	16	neryl acetate (39.6%), α-curcumene (7.6%)
*Myrtus communis*	1.50 (32)	0.10 (512)	16	α-pinene (52.9%), 1,8-cineole (20.6%)
*Cedrus atlantica*	50.00 (1)	6.00 (8)	8	α-pinene (55%), himachalol (8.3%)
*Lavandula stoechas*	1.50 (32)	0.20 (256)	8	fenchone (34.9%), camphor (28.9%)
*Calamintha nepeta* ssp. *nepeta*	0.80 (64)	0.20 (256)	4	pulegone (49.0%), menthone (21.5%)
*Citrus clementina*	1.50 (32)	0.40 (128)	4	sabinene (31.4%), linalool (20.4%)
*Cymbopogon citratus*	0.80 (64)	0.20 (256)	4	geranial (44.0%), neral (31.1%)
*Foeniculum vulgare*	0.80 (64)	0.20 (256)	4	(*E*)-anethole (75.7%), limonene (8.5%)
*Xanthoxylum armatum*	0.40 (128)	0.20 (256)	2	linalool (52.9%), limonene (15.0%)

**Table 2:** Antimicrobial and anti-QS assays of pulegone, *cis-cis-p-menthenolide* and mintlactone.

	Pulegone	*cis-cis-p-*Menthenolide	Mintlactone
Chemical structure	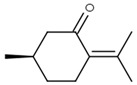	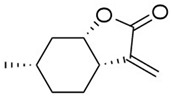	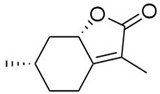
MIC value (mg·mL^−1^)/MIC dilution	0.40 (128)	3.00 (16)	3.00 (16)
MQSIC value (mg·mL^−1^)/MQSIC dilution	0.10 (512)	0.05 (1024)	0.40 (128)
MQSIC/MIC ratio	4	64	8

## References

[1] Waters C.M., Bassler B.L. (2005). Quorum sensing: Cell-to-cell communication in bacteria. Annual Review of Cell and Developmental Biology.

[2] Asfour H.Z. (2017). Antiquorum sensing natural compounds. J. Microsc. Ultrastruct..

[3] McLean R.J.C., Pierson L.S., Fuqua C. (2004). A simple screening protocol for the identification of quorum signal antagonists. J. Microbiol. Methods.

[4] Dessaux Y., Elmerich C., Faure D. (2004). La violacéine: Une molécule d’intérêt biologique, issue de la bactérie tellurique Chromobacterium violaceum. Rev. Méd. Interne.

[5] Zinger-Yosovich K., Sudakevitz D., Imberty A., Garber N.C., Gilboa-Garber N. (2006). Production and properties of the native Chromobacterium violaceum fucose-binding lectin (CV-IIL) compared to homologous lectins of Pseudomonas aeruginosa (PA-IIL) and Ralstonia solanacearum (RS-IIL). Microbiology.

[6] Zins M.M., Zimprich C.A., Petermann S.R., Rust L. (2001). Expression and partial characterization of an elastase from Chromobacterium violaceum. Vet. Microbiol..

[7] Durán N., Justo G.Z., Durán M., Brocchi M., Cordi L., Tasic L., Castro G.R., Nakazato G. (2016). Advances in Chromobacterium violaceum and properties of violacein-Its main secondary metabolite: A review. Biotechnol. Adv..

[8] Chernin L.S., Winson M.K., Thompson J.M., Haran S., Bycroft B.W., Chet I., Williams P., Stewart G. (1998). Chitinolytic activity in Chromobacterium violaceum: Substrate analysis and regulation by quorum sensing. J. Bacteriol..

[9] De Almeida D.F., Hungria M., Guimaraes C.T., Antonio R.V., Almeida F.C., de Almeida L.G.P., de Almeida R., Alves-Gomes J.A., Andrade E.M., Araripe J. (2003). The complete genome sequence of Chromobacterium violaceum reveals remarkable and exploitable bacterial adaptability. Proc. Natl. Acad. Sci. USA.

[10] McClean K.H., Winson M.K., Fish L., Taylor A., Chhabra S.R., Camara M., Daykin M., Lamb J.H., Swift S., Bycroft B.W. (1997). Quorum sensing and Chromobacterium violaceum: Exploitation of violacein production and inhibition for the detection of *N*-acylhomoserine lactones. Microbiology.

[11] Morohoshi T., Kato M., Fukamachi K., Kato N., Ikeda T. (2008). *N*-Acylhomoserine lactone regulates violacein production in Chromobacterium violaceum type strain ATCC 12472. FEMS. Microbiol. Lett..

[12] Kothari V., Sharma S., Padia D. (2017). Recent research advances on Chromobacterium violaceum. Asian Pac. J. Trop. Med..

[13] Champ M.A. (2003). Economic and environmental impacts on ports and harbors from the convention to ban harmful marine anti-fouling systems. Mar. Pollut. Bull..

[14] Yebra D.M., Kiil S., Dam-Johansen K. (2004). Antifouling technology—Past, present and future steps towards efficient and environmentally friendly antifouling coatings. Prog. Org. Coat..

[15] Kalemba D., Kunicka A. (2003). Antibacterial and antifungal properties of essential oils. Curr. Med. Chem..

[16] Orhan F., Sekerel B.E., Kocabas C.N., Sackesen C., Adalioglu G., Tuncer A. (2003). Complementary and alternative medicine in children with asthma. Ann. Allergy Asthma Immunol..

[17] Bassole I.H.N., Juliani H.R. (2012). Essential Oils in Combination and Their Antimicrobial Properties. Molecules.

[18] Azzimonti B., Cochis A., Beyrouthy M.E., Iriti M., Uberti F., Sorrentino R., Landini M.M., Rimondini L., Varoni E.M. (2015). Essential Oil from Berries of Lebanese Juniperus excelsa M. Bieb Displays Similar Antibacterial Activity to Chlorhexidine but Higher Cytocompatibility with Human Oral Primary Cells. Molecules.

[19] Scotti F., Decani S., Sardella A., Iriti M., Varoni E.M., Lodi G. (2018). Anti-inflammatory and wound healing effects of an essential oils-based bioadhesive gel after oral mucosa biopsies: Preliminary results. Cell. Mol. Biol..

[20] De Kievit T.R., Iglewski B.H. (2000). Bacterial quorum sensing in pathogenic relationships. Infect. Immun..

[21] Defoirdt T., Boon N., Bossier P. (2010). Can Bacteria Evolve Resistance to Quorum Sensing Disruption?. PLoS Pathog..

[22] Kerekes E.-B., Deák É., Takó M., Tserennadmid R., Petkovits T., Vágvölgyi C., Krisch J. (2013). Anti-biofilm forming and anti-quorum sensing activity of selected essential oils and their main components on food-related micro-organisms. J. Appl. Microbiol..

[23] Luciardi M.C., Blázquez M.A., Cartagena E., Bardón A., Arena M.E. (2016). Mandarin essential oils inhibit quorum sensing and virulence factors of Pseudomonas aeruginosa. LWT Food Sci. Technol..

[24] Djabou N., Lorenzi V., Guinoiseau E., Andreani S., Giuliani M.-C., Desjobert J.-M., Bolla J.-M., Costa J., Berti L., Luciani A. (2013). Phytochemical composition of Corsican Teucrium essential oils and antibacterial activity against foodborne or toxi-infectious pathogens. Food Control..

[25] Guinoiseau E., Luciani A., Serra D.D.R., Quilichini Y., Berti L., Lorenzi V. (2015). Primary Mode of Action of *Cistus ladaniferus* L. Essential Oil Active Fractions on Staphylococcus aureus Strain. Adv. Microbiol..

[26] Li L., Li Z.-W., Yin Z.-Q., Wei Q., Jia R.-Y., Zhou L.-J., Xu J., Song X., Zhou Y., Du Y.-H. (2014). Antibacterial activity of leaf essential oil and its constituents from Cinnamomum longepaniculatum. Int. J. Clin. Exp. Med..

[27] Kazemi M., Mousavi E., Kharestani H. (2012). Chemical Compositions and Antimicrobial Activities of Essential Oils of *Varthemia persica*, *Foeniculum vulgare* and *Ferula lycia*. Curr. Res. Bacteriol..

[28] Onawunmi G.O. (1989). Evaluation of the antimicrobial activity of citral. Lett. Appl. Microbiol..

[29] Agnaniet H., Makani T., Bikanga R., Obame L.C., Lebibi J., Menut C. (2009). Chemical Composition and Antimicrobial Activity of the Essential Oils of Leaves, Roots and Bark of Glossocalyx staudiii. Nat. Prod. Commun..

[30] Sokovic M., Glamoclija J., Marin P.D., Brkic D., van Griensven L.J.L.D. (2010). Antibacterial Effects of the Essential Oils of Commonly Consumed Medicinal Herbs Using an In Vitro Model. Molecules.

[31] Alvarez M.V., Moreira M.R., Ponce A. (2012). Antiquorum sensing and antimicrobial activity of natural agents with potential use in food. J. Food Saf..

[32] Olivero-Verbel J., Barreto-Maya A., Bertel-Sevilla A., Stashenko E.E. (2014). Composition, anti-quorum sensing and antimicrobial activity of essential oils from Lippia alba. Braz. J. Microbiol..

[33] Salido S., Altarejos J., Nogueras M., Saánchez A., Luque P. (2003). Chemical Composition and Seasonal Variations of Rosemary Oil from Southern Spain. J. Essent. Oil Res..

[34] Swords G., Hunter G. (1978). Composition of Australian Tea Tree Oil (melaleuca-Alternifolia). J. Agric. Food Chem..

[35] Duru M.E., Öztürk M., Uğur A., Ceylan Ö. (2004). The constituents of essential oil and in vitro antimicrobial activity of *Micromeria cilicica* from Turkey. J. Ethnopharmacol..

[36] Sutour S., Bradesi P., de Rocca-Serra D., Casanova J., Tomi F. (2008). Chemical composition and antibacterial activity of the essential oil from *Mentha suaveolens* ssp. insularis (Req.) Greuter. Flavour Fragr. J..

[37] Azeredo J., Azevedo N.F., Briandet R., Cerca N., Coenye T., Costa A.R., Desvaux M., Di Bonaventura G., Hebraud M., Jaglic Z. (2017). Critical review on biofilm methods. Crit. Rev. Microbiol..

[38] Sutour S., Bradesi P., Luro F., Casanova J., Tomi F. (2015). Germacra-1,5-dien-4α-ol in *Fortunella* sp. leaf oils. Flavour Fragr. J..

[39] Sutour S., Therrien B., von Reuss S.H., Tomi F. (2018). Halogenated C15 Acetogenin Analogues of Obtusallene III from a *Laurenciella* sp. Collected in Corsica. J. Nat. Prod..

[40] Adonizio A.L., Downum K., Bennett B.C., Mathee K. (2006). Anti-quorum sensing activity of medicinal plants in southern Florida. J. Ethnopharmacol..

[41] Pellegrini M.C., Alvarez M.V., Ponce A.G., Cugnata N.M., De Piano F.G., Fuselli S.R. (2014). Anti-quorum sensing and antimicrobial activity of aromatic species from South America. J. Essent. Oil Res..

